# Risk factors for poor outcome in patients with osmotic demyelination syndrome

**DOI:** 10.1186/cc10750

**Published:** 2012-03-20

**Authors:** MA Peters, JG Van der Hoeven, C Hoedemaekers

**Affiliations:** 1 Canisius Wilhemina Hospital, Nijmegen, the Netherlands; 2 Radboud University Nijmegen Medical Centre, Nijmegen, the Netherlands

## Introduction

The osmotic demyelination syndrome (ODS) is a devastating complication of rapid correction of hyponatremia. The objective of this study was to identify prognostic factors that determine outcome in patients with ODS.

## Methods

We performed a literature search using MEDLINE and Embase. Case reports or case series were eligible for this study in cases of: (1) hyponatremia defined as a serum sodium ≤130 mEq/l on hospital admission or thereafter, but preceding the clinical signs of ODS; (2) a clear diagnosis of ODS, confirmed by MRI scanning or pathology; and (3) a description of patient outcome. We defined a favourable outcome as a Glasgow Outcome Score >3 or a Modified Rankin Scale <4.

## Results

A total of 120 manuscripts were identified describing 125 cases: 86/125 (69%) had a favourable outcome. Mean age in the favourable outcome group was 44.7 ± 14.4 years versus 52.3 ± 13.6 years in the poor outcome group (*P *= 0.006). The ODS was exclusively pontine in 44/125 (35%), extrapontine in 34/125 (37%) and combined pontine and extrapontine in 47/125 (37%) of cases. The anatomical localisation of the lesion was not associated with outcome (*P *= 0.64). Forty-two out of 125 (34%) of cases were associated with alcohol abuse, 14/125 (11%) with malnutrition, 26/125 (21%) with use of diuretics and 9/125 (7%) with use of psychoactive medication; none of these characteristics were significantly related to outcome. The sodium concentration on admission was 107.3 ± 9.6 in the patients with a favourable outcome versus 108.4 ± 9.4 in the patients with a poor outcome (*P *= 0.54). The speed of sodium correction was 1.12 ± 1.6 mmol/hour versus 1.16 ± 0.9 mmol/hour respectively in the favourable and poor outcome cases (*P *= 0.19). The highest sodium concentration after correction was significantly higher in the patients with a poor outcome (139.0 ± 9.3 vs. 134.0 ± 7.3, *P *= 0.003). Serum osmolality, and concentrations of potassium, chloride, creatinin and glucose were comparable between the outcome groups. The development of tetraparesis (55/125 (44%), *P *= 0.02) or a decreased level of consciousness (58/125 (46%), *P *< 0.001) were associated with a poor outcome. In contrast, mutism or dysarthria (82/125 (66%), *P *= 0.002), tremors (29/125 (23%), *P *= 0.001) or ataxia (58/125 (46%), *P *< 0.001) were associated with a favourable outcome.

## Conclusion

The highest serum sodium concentration during sodium correction rather than the speed of sodium correction or severity of the hyponatremia is a determinant of outcome in patients with ODS. The development of tetraparesis and decreased consciousness are associated with a poor outcome in these patients.

